# Integration of Hierarchical Micro-/Nanostructures in a Microfluidic Chip for Efficient and Selective Isolation of Rare Tumor Cells

**DOI:** 10.3390/mi10100698

**Published:** 2019-10-14

**Authors:** Shunqiang Wang, Younghyun Cho, Xuanhong Cheng, Shu Yang, Yi Liu, Yaling Liu

**Affiliations:** 1 Department of Mechanical Engineering and Mechanics, Lehigh University, Bethlehem, PA 18015, USA; sqwang1990@gmail.com; 2 Department of Materials Science and Engineering, University of Pennsylvania, Philadelphia, PA 19104, USA; yhcho@sch.ac.kr (Y.C.); shuyang@seas.upenn.edu (S.Y.); 3 Department of Materials Science and Engineering, Lehigh University, Bethlehem, PA 18015, USA; xuc207@lehigh.edu; 4 School of Engineering, Dali University, Dali 671000, Yunnan, China; 5 Department of Bioengineering, Lehigh University, Bethlehem, PA 18015, USA

**Keywords:** microfluidics, nanoparticles, circulating tumor cell (CTC) isolation

## Abstract

Circulating tumor cells (CTCs) are important clinical markers for both cancer early diagnosis and prognosis. Various techniques have been developed in the past decade to isolate and quantify these cells from the blood while microfluidic technology attracts significant attention due to better controlled microenvironment. When combined with advanced nanotechnologies, CTC isolation performance in microfluidic devices can be further improved. In this article, by extending the wavy-herringbone concept developed earlier in our team, we prepared a hierarchical microfluidic chip by introducing a uniform coating of nanoparticles with anti-epithelial cell adhesion molecule (EpCAM) on wavy microgrooves. This hierarchical structured platform not only maintains the capture purity of the wavy-herringbone structure but improves the capture efficiency thanks to the larger surface area to volume ratio brought by nanoparticles. Our results demonstrated a capture efficiency of almost 100% at a low shear rate of 60/s. Even at a higher shear rate of 400/s, the hierarchical micro/nanostructures demonstrated an enhancement of up to ~3-fold for capture efficiency (i.e., 70%) and ~1.5-fold for capture purity (i.e., 68%), compared to wavy-herringbone structures without nanoparticle coating. With these promising results, this hierarchical structured platform represents a technological advancement for CTC isolation and cancer care.

## 1. Introduction

Tumors are among the leading causes of deaths across the world. Tumor detection, especially in the early stage, is of great interest to both researchers and clinicians. Conventional imaging techniques such as MRI may be used for initial confirmation of cancer occurrence while malignancy still requires an invasive biopsy [1]. However, the discomfort and risk of infection brought by most invasive biopsies lay a hurdle for general acceptance. Liquid biopsy is appealing for cancer diagnosis due to its non-invasiveness and easy sampling procedure. Several cancer biomarkers have been identified [2,3,4,5], among which circulating tumor cells (CTCs) have attracted a lot of attention recently. CTCs are originated from primary tumors and circulating in the blood vessel during metastasis. Recent studies have revealed that the number of CTCs can be used for early cancer detection [6] and cancer prognosis [7,8]. However, detecting CTCs is inherently challenging due to the cell rarity, i.e., 1-100 CTCs per 1 mL blood versus billions of normal blood cells. Various techniques have been developed to overcome the challenge, taking advantage of unique physical properties (e.g., size [9,10], dielectricity [11,12], and deformability [13,14]) and surface biochemistry [15,16,17] of CTCs. Immunoaffinity separation uses specific antibody-antigen interactions to either capture CTCs (positive isolation [15,16]) or depletes [18,19] white blood cells (WBCs, negative isolation). Although promising results with high capture efficiencies have been achieved in various platforms [15,16,20,21], the capture purity or specificity remains to be improved.

The advancement of micro- and nanofabrication techniques enables the design of new smart structures to enhance the performance of CTC microfluidic chips. A hallmark microfluidic device with grooved herringbone (HB) structures [22] was shown to isolate CTCs with a capture efficiency up to 93% and a purity of 14% by stretching the HB structures and disturbing the flow streamlines to increase cell-surface interactions. Various derivatives of the HB chip [20,23,24] were developed subsequently, for example, by incorporation of nanostructures, including nanopillars [25] and nanovelcro [26], into microfluidics to increase the overall surface area and enhance the cell-surface interactions. These hierarchical structures presented a better capture efficiency and their clinical utilities were demonstrated on patients of various cancer phenotypes [26,27,28]. However, one main limitation of the grooved-HB chip is non-uniform shear stress distribution in the channel, thus trapping WBCs in regions with extremely low shear stress and sacrifices the purity. To overcome this limitation, we have recently developed a wavy-HB chip where the smooth groove edges eliminate regions with extremely low shear stress. The wavy-HB chips showed a capture efficiency up to 85% and a purity up to 39.4% [24,29].

Leveraging our success with the wavy-HB chips and knowledge that nanostructures with a diameter of 100 nm enhance CTC capture [30], here we combined the two features by coating the wavy-HB microstructure with nanoparticles (NPs) in this study. This hierarchical structure reflects advantages from both microscale and nanoscale: by constructing the wavy-HB microstructures, the chip preserves a high purity by eliminating the extremely low-shear regions (demonstrated in an earlier study [31]), by integrating NPs, the chip presents a high capture efficiency due to both HB induced vortex effect and NP induced enhanced cell-surface interactions. As follows, the fabrication steps to integrate the wavy-HB microstructures and NPs were described, followed by scanning electron microscopy (SEM) characterization. The working mechanism of the microfluidic chip was first proposed. Finally, the tumor cell capture tests were performed and the results were compared with other designs reported in literature.

## 2. Method and Materials

### 2.1. Fabrication and Surface Functionalization of the Hierarchical CTC Chip 

**Figure 1:**
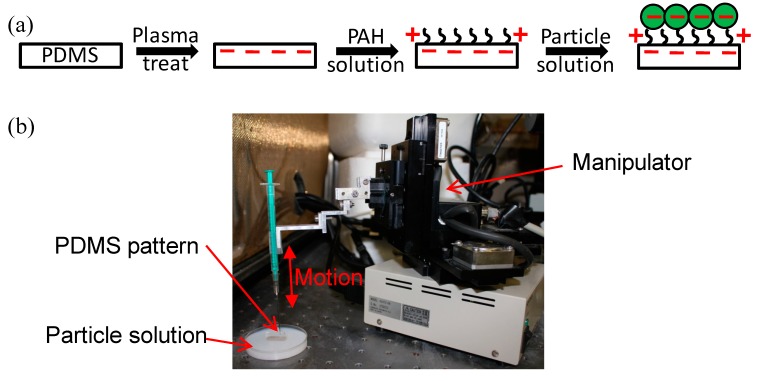
(**a**) Flow chart of nanoparticle (NP) deposition on the wavy-herringbone (HB) polydimethylsiloxane (PDMS) device. (**b**) Experimental setup for NP deposition. The PDMS device was slowly withdrawn from a particle suspension using a home-build manipulator and nanoparticles were deposited into a thin film on the PDMS.

The hierarchical microfluidic chip was fabricated in two steps, as shown in Figure 1. First, a polydimethylsiloxane (PDMS) slab with micron sized wavy-HB structures was made by using the reflow process reported earlier [25]. Briefly, an SU8 master with sharp grooved-HB was first fabricated by photolithography, followed by replication to a PDMS mold. A second SU8 pattern was then replicated through capillary force lithography by pressing the PDMS mold on an uncured photoresist layer at 95 °C. After cooling down to room temperature and removing the PDMS mold, the sharp grooved-HB structures were melted into wavy-HB structures by baking at 55 °C. The wavy-HB SU8 master was then treated with 1H,1H,2H,2H-perfluorodecyltrichlorosilane (FDTS, Alfa Aesar, Ward Hill, MA, USA) and used for PDMS replication. APDMS cover layer was made through the traditional soft lithography method, followed by punching the inlet and outlet holes.

To ensure a uniform coating of NPs on the wavy-HB structures, NPs were deposited via electrostatic interactions between oppositely charged polymers [32]. First, negative charges were introduced to the surface of the wavy-HB structures treated with O_2_ plasma. The PDMS slab was then immersed in a solution of 0.01 mol/L poly(arylaminehydrochloride) (PAH, Sigma Aldrich, St. Louis, MO, USA) for 5 min to form a layer of positively charged polymers. After rinsing with DI water to remove the excess PAH, negatively charged silica NPs with a diameter of 100 nm (nanoComposix, San Diego, CA, USA) were deposited by immersing the PDMS slab in 1% NP ethanol solution for 5 s. To control the deposition consistency for each deposition, an automated manipulator was used to fix the pulling velocity at 5 mm/min [33]. The deposited PDMS slab was dried overnight under an enclosed environment of ethanol to reduce particle clumping due to evaporation [34,35]. The resulting hierarchical structures were then characterized by scanning electron microscopy (SEM). Lastly, the hierarchical PDMS slab was assembled with the PDMS cover layer to make a microfluidic chip.

For the test of cancer cell capture, microfluidic chips were chemically functionalized with anti-EpCAM following a standard protocol [25]. Briefly, 3-mercaptopropyl trimethoxysilane (MPTS, Sigma Aldrich) was covalently linked with hydroxyl groups on the PDMS surface introduced by oxygen plasma treatment. N-γ-maleimidobutyryl-oxysuccinimide ester (GMBS) was then reacted with MPTS, followed by a coating of NeutrAvidin. Within 4 hours of the cell capture test, biotinylated anti-EpCAM was introduced to the microfluidic device. Regions uncovered by anti-EpCAM were blocked with 5% bovine serum albumin to reduce non-specific cell capture.

### 2.2. Preparation of Cell Samplesand Cell Capture

In this study, blood spiked with HCT-116 cells were used to model CTC captured from patients with colon cancer. Cells were cultured following a standard protocol by routinely feeding the cells with a medium comprised of Dulbecco’s Modified Eagle’s Medium, 1% penicillin/streptomycin antibiotics, and 10% fetal bovine serum. Upon 70–80% confluency, cells were trypsinized and diluted to 10^3^–10^5^ /mL in either a phosphate buffered saline (PBS) buffer solution containing 4 mg/mL alginate or healthy whole blood purchased from Innovative Research.

The cell suspension was injected into microfluidic chips at prescribed flow rates ranging from 580 μL/hr to 3600 μL/hr, which covers the range commonly used in the literature [16,36]. A PBS buffer solution was injected right after cell capture to remove all suspended cells, followed by a fixation process using 4% paraformaldehyde. To identify the captured cells, cells were treated with 0.2% Triton X-100 and stained with a cocktail solution of 4′,6-diamidino-2-phenylindole (DAPI), anti-cytokeratin fluorescein isothiocyanate (FITC) and CD45 PE. Cells that were DPAI positive, cytokeratin positive and CD45 negative were identified as tumor cells, while the ones that were DAPI positive, cytokeratin negative and CD45 positive were treated as WBCs. Meanwhile, only cells with morphology integrity were counted.

To identify cell viability, a cocktail solution containing calcein-AM and ethidium homodimer (EthD-1) was used to perform a LIVE/DEAD assay. Cells were treated as viable if they were calcein-AM positive and EthD-1 negative, while all others were treated as dead.

## 3. Results and Discussions

### 3.1. Working Mechanism

**Figure 2:**
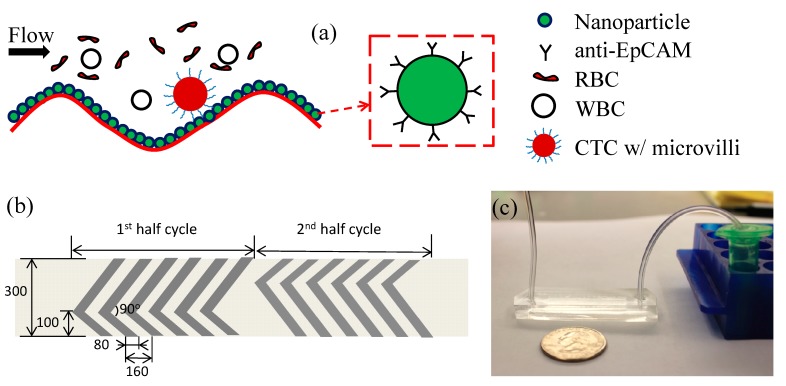
(**a**) Concept illustration of cancer cell capture in a microfluid chip with hierarchical micro/nanostructures. NPs coated with anti-epithelial cell adhesion molecule (EpCAM) are deposited on the wavy-HB pattern. (**b**) Illustrative geometry of a cycle of the wavy-HB pattern. Unit: μm. (**c**) Photographic image of the test setting for the hierarchical circulating tumor cells (CTCs) capture chip.

Deriving from the wavy-HB chip developed by us earlier [25], the hierarchical CTC chip contained NPs deposited on the wavy-HB pattern, as shown in Figure 2a. As a proof-of-concept study, the geometry of the wavy-HB pattern was the same as that in the previous work [16], where the wavelength is 160 μm, wave amplitude is 22.5 μm, short/long arms are 100 μm/200 μm, intersection angle of short/long arm is 90° and channel height is 50 μm. Based on the geometry guide of nanostructures to optimize cancer cell capture [30], NPs with a diameter of 100 nm were used.

Both the numerical and experimental analysis demonstrated high capture efficiency using the wavy-HB pattern [24,31], due to significant vortex effect and large cell-surface contact area. Furthermore, high purity can be achieved by eliminating the extremely low shear rate regions in conventional HB structures with sharp corners. The NP coated wavy-HB patterns inherit these advantages. Since the diameter of the NPs (100 nm) is ~1/500 of the micropattern wavelength, the existence of the NPs rarely influences the flow domain. Furthermore, the integration of NPs allows for the further increase of the cell-surface interaction area due to their large surface-to-volume ratio. Assuming a coverage of a monolayer of 100 nm NPs on the wavy-HB pattern, the surface area can be doubled compared to a plain surface, which in turn will enhance cell-surface interactions and cell capture. The increased surface area is not expected to promote WBC capture, however, as the surface is passivated by bovine serum albumin (BSA).

### 3.2. Characterization of Hierarchical Micro/nanostructures

**Figure 3:**
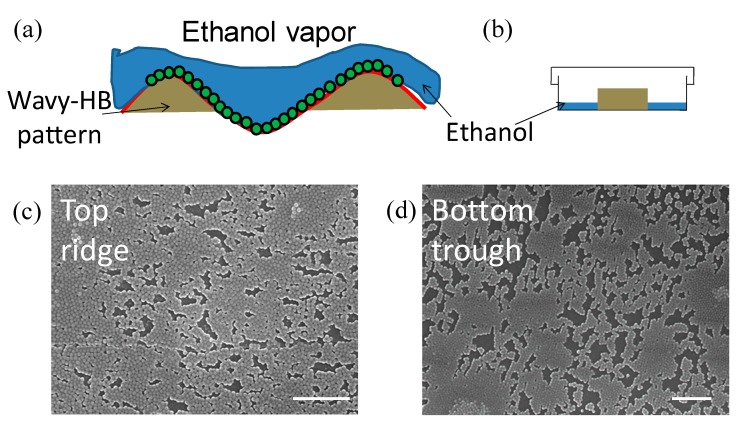
(**a**) Illustrative image of the NP deposition under the (**b**) improved evaporation setting. Scanning electron microscopy (SEM) images of deposited NPs on the (**c**) ridge and (**d**) in the trough after the deposition process. Scale bar: 2 μm.

As shown in Figure 1a, the PDMS slab is dried after withdrawing from the particle solution. It is noticed that if directly exposed to air and dried overnight, NPs always accumulate on the ridge leading to non-uniform coating (Figure S1a,b). This is a result of evaporation induced- convection pulling NPs from the trough to the ridge [34,35], as illustrated in Figure S1c. In other words, the ethanol film evaporates fast in the ridge as it is thin compared to that in the trough. As a result, it can trigger a convective flow with NPs moving from the trough to the ridge. To reduce this effect, the PDMS slab is dried in a petri dish containing a small amount of ethanol solution, illustrated in Figure 3a,b. Although it increases the time for the drying process, ethanol vapor reduces the above-mentioned evaporation induced convection, leading to a more uniform coating of NPs over the whole wavy-HB pattern, as shown in Figure 3c,d.

### 3.3. Cancer Cell Capture

**Figure 4:**
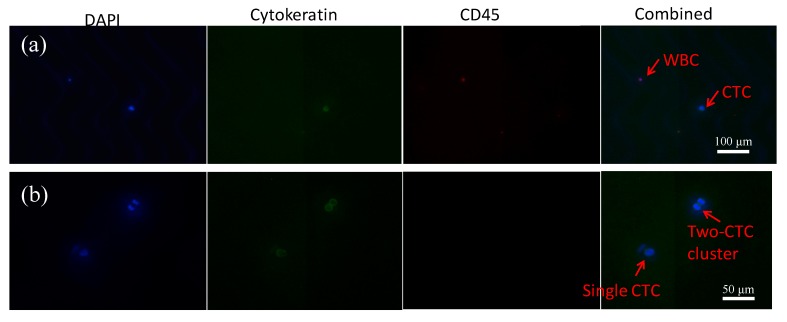
(**a**) Fluorescent images of a single cancer cell and a single WBC captured in the device and stained by DAPI, Cytokeratin-FITC, and CD45-PE. (**b**) Fluorescent images of a single cancer cell and a cancer cell cluster by using the same stain.

To evaluate the device performance, HCT-116 cancer cells were spiked into both PBS buffer and healthy whole blood. After cell capture, a standard fluorescent staining protocol was used to differentiate captured cancer cells and WBCs, which were then counted to calculate the capture efficiency and purity. Typical fluorescent images of captured cancer cells and WBCs are shown in Figure 4a,b. Furthermore, it was noticed that the hierarchical wavy-HB chip could capture both single cancer cells and cell clusters, which were shown to better correlate to cancer prognostics [8].

**Figure 5:**
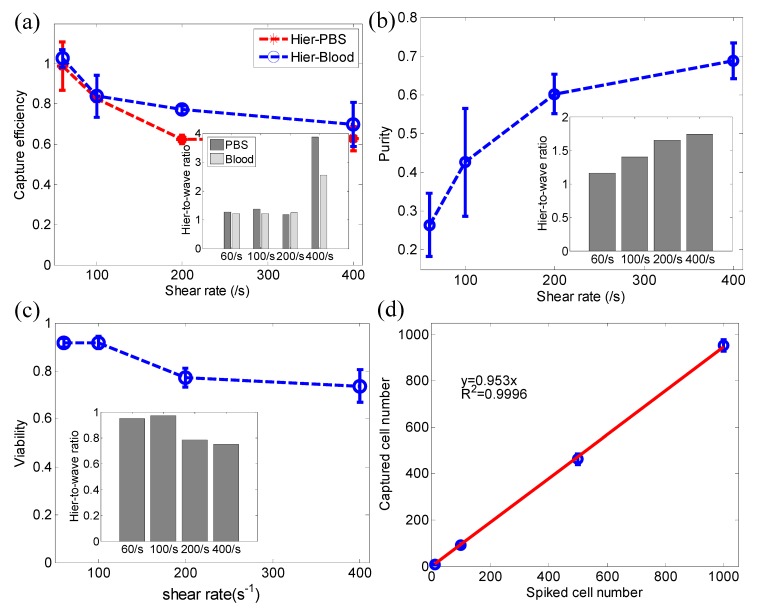
Performance of the hierarchical wavy-HB chip in comparison to the pure wavy-HB chip. (**a**) Capture efficiency vs. shear rate in the hierarchical wavy-HB chip from both PBS buffer and whole blood. (**b**) Purity vs. shear rate in the hierarchical wavy-HB chip. (**c**) Viability vs. shear rate in the hierarchical wavy-HB chip. (**d**) Captured cell numbers in experiments with PBS spiked with a different number of CTCs. The insets in (a)-(c) show the ratio between the hierarchical wavy-HB chip and the pure wavy-HB chip. Error bars indicate the standard deviation from three independent experiments.

A side-by-side comparison between the proposed hierarchical wavy-HB chip and the conventional wavy-HB chip is presented in Figure 5a. The overall trend of capture efficiency over the shear rate in the hierarchical wavy-HB chip agrees well with that for the pure wavy-HB chip in both PBS and whole blood. The decrease with shear rate is mainly due to increased shear detachment. However, the hierarchical wavy-HB chip outweighs the pure wavy-HB chip in capture efficiency (inset of Figure 5a). Especially at a high shear rate of 400/s, the enhancement is more than 2 times in whole blood (69.8% vs. ~27.3%). This enhancement was speculated to arise from increased total surface area brought by the integration of NPs, which agrees well with the findings in other works in literature [37,38]. Figure 5b shows that purity is enhanced with increasing shear rates. Interestingly, the inset in Figure 5b shows that the hierarchical wavy-HB chip owns a better purity performance compared to the pure wavy chip. This finding can be explained by our selection of optimized NP size (100 nm) which leads to a larger NP-CTC adhesion force [30] while the non-specific NP-WBC is not affected. Based on results shown in Figure 5a,b, the hierarchical wavy-HB chip can lead to better capture efficiency and purity than the pure wavy-HB chip. This is extremely important in CTC post-analysis, such as DNA analysis of isolated CTCs, where both high capture efficiency and high purity are required. In this case, the hierarchical wavy-HB chip can serve as a suitable candidate to be tested at high shear rates like 200/s and 400/s. The hierarchical wavy-HB chip also presents cells with high viability, as shown in Figure 5c. The potential clinical application was also demonstrated by investigating capture using CTCs spiked at different concentrations ranging from 10 to 1000 per mL whole blood at a shear rate of 60/s (see Figure 5d). Future efforts to facilitate post-analysis of isolated CTCs could be readily achieved by using magnetic nanoparticles, which allow a sequential capture and release of CTCs. Also, with an increasing clinical need to isolate EpCAM- cell lines, the proposed platform can be extended for those applications by modifying the surface coated antibody from anti-EpCAM to other antibodies corresponding to biomarkers of interest.

**Figure 6:**
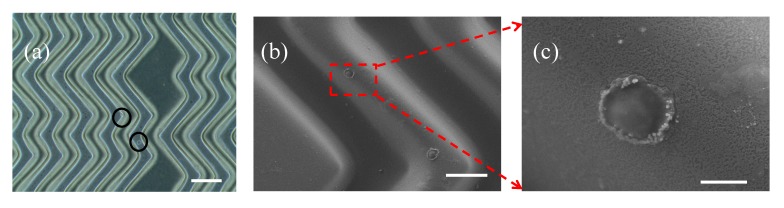
(**a**) Microscopic image of captured cancer cells on the hierarchical wavy-HB pattern. Scale bar: 160 μm. (**b**) SEM image of captured cancer cells on the pattern. Scale bar: 50 μm. (**c**) A zoom-in image reveals details of the interaction between a cancer cell and the NPs covered wavy pattern. Scale bar: 8 μm.

Figure 6a shows a typical microscopic image of captured cancer cells in the hierarchical wavy-HB chip. Furthermore, SEM images were taken to reveal the details including cell morphology and cell-surface interactions, as shown in Figure 6b,c. These images confirm that NPs remained on the wavy-HB pattern after cell capture. The projected cell-surface contact area was calculated to be an average of 101.7 μm^2^ which is calculated based on SEM images. This number is comparable to the previously reported value (~110 μm^2^) of cancer cells spread on a bare wafer surface and smaller than that (~280 μm^2^) on a monolayer of NPs 120 nm in diameter [30]. The result is expected since prior work used a one-hour incubation to allow cancer cells to spread over the substrate while in this study, cancer cells were fixed right after the sample flow. With the increased effective contact area due to the NPs, cancer cells were captured more effectively compared to the bare wavy-HB surface.

## 4. Conclusions

A microfluidic CTC isolation chip with hierarchical micro/nanostructures were developed. By extending the concept of the wavy-HB chip, we show that the integration of nanoparticles with wavy-HB chip enhanced CTC capture due to the increased overall surface area. A previously developed particle deposition approach was used to fabricate the hierarchical patterns. In comparison to the conventional wavy-HB chip and the hallmark grooved-HB chip, the wavy-HB chip with hierarchical micro/nanostructures demonstrated a capture efficiency up to almost 100% at a low shear rate (i.e., 60/s) and a high purity performance (up to ~70% and only ~680 WBCs per 1 mL blood). SEM images were also used to further characterize the interaction between captured cancer cells and micro/nanostructures.

## Supplementary Materials

The following are available online at https://www.mdpi.com/2072-666X/10/10/698/s1, Figure S1: SEM images of deposited nanoparticles (NPs) on (**a**) top ridge and (**b**) bottom trough after the deposition process. (**c**) Illustrative image of the NP convection induced by ethanol evaporation if exposed directly to air. More particles are thus deposited on the top ridge and less particles on bottom trough. Scale bar: 2 μm.
