# Supplementary material for: Integration of Hierarchical Micro-/Nanostructures in a Microfluidic Chip for Efficient and Selective Isolation of Rare Tumor Cells
Source: Micromachines (Basel). 2019 Oct 14;10(10):698. doi: 10.3390/mi10100698 (PMC6843196; doi:10.3390/mi10100698)
Supplement: Supplementary file 1 [file micromachines-10-00698-s001.pdf]

micromachines

Integration of Hierarchical Micro-/Nanostructures in a Microfluidic Chip for Efficient and Selective Isolation of Rare Tumor Cells

Shunqiang Wang, Younghyun Cho, Xuanhong Cheng, Shu Yang, Yi Liu and Yaling Liu

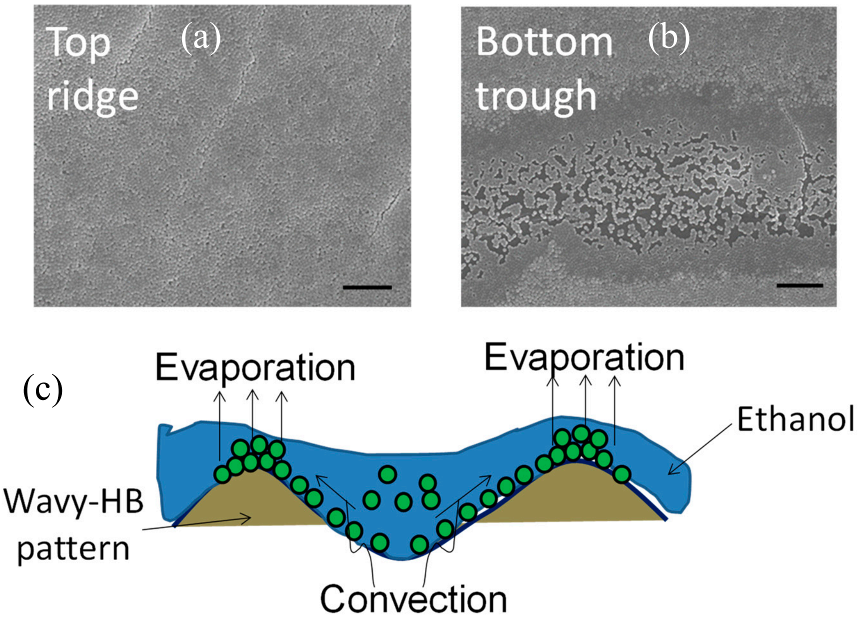

Figure S1. SEM images of deposited nanoparticles (NPs) on (**a**) top ridge and (**b**) bottom trough after the deposition process. (**c**) Illustrative image of the NP convection induced by ethanol evaporation if exposed directly to air. More particles are thus deposited on the top ridge and less particles on bottom trough. Scale bar: 2 μm.
